# Supplementary material for: TBC9, an essential TBC-domain protein, regulates early vesicular transport and IMC formation in Toxoplasma gondii
Source: Commun Biol. 2024 May 18;7:596. doi: 10.1038/s42003-024-06310-6 (PMC11102469; doi:10.1038/s42003-024-06310-6)
Supplement: Supplementary file 2 — Supplementary Information [file 42003_2024_6310_MOESM2_ESM.pdf]

Supplementary Information

TBC9, an essential TBC-domain protein, regulates early vesicular transport and IMC formation in *Toxoplasma gondii*

Ming Sun[a], Tao Tang[a], Kai He[a] and Shaojun Long[a,#]

[a]National Key Laboratory of Veterinary Public Health Safety and College of Veterinary Medicine, China Agricultural University, Beijing 100193, China

[#]Correspondence: Email: LongS2018@163.com

This Supplementary Information includes
Supplementary Figures 1-7 and Legends;
Supplementary Table 1;
Supplementary Reference.

Supplementary Figures 1-7 and Legends

```
TgTBC1  : MEEIWKDVERTF----ADRA-LFC---RDATRKALQRILFTWSRQNPDV---SYKQGMNELLAIL--FLICV :  207
TgTBC2  : -DTCDLLEPRRH----YDVL--------------GRILFVYAKVNPGI---RYVQGMNELLAPI--YYVIM : 1170
TgTBC3  : GPIIARDINRTF----PKHI-LFRD--HQKGQQALFNVLKAYAIFNPDV---GYCQGMGFLSGIL--LMYM- :  208
TgTBC4  : -RQLRVDIPRTH----SGRL-FFS---HPRIQACMERALFLWAVKNPAS---GYVQGMNDLITPF--LSVFL :  181
TgTBC5  : -LLANDDFSQCY----EHHD-------SRYGRRQLRCLLQALFAQNSGMS--FSARGLDAIAAPL-QLLYMN :  208
TgTBC6  : SGGAASDKARQPV---PLHV-YRRR--TESGKEKARRLLWCLNALHGTV---EFCPVLPPLTCVL--LLYF- : 1199
TgTBC7  : -RVIRSDVERTR----ASLA-FYR---DADSRAWMEKLLTNYC-KTCHI---KYKQGLNELLAPF--LYLKG :  123
TgTBC8  : -EQIELDLLRTF----PTNR-RFRG--KAGGVADLRQVLWAFAAYKPKI---NYCQSMNFLAATL--LLFM- :  233
TgTBC9  : -RIFVLDAERTFK--DPKH------------REQMVSVLQS---LWPELQ--DYHQGLGFLVAFL--LLYLP :  115
TgTBC10 : FALIMIDVPRTF----PDVE-VF----DKDAQALLCRNLNAFANIHPEV---GYCQGMNFIAGLL--LLVSS :  211
TgTBC12 : KEQIGKDVARSMH--GPGEV-RRRH--ATCLRGTLQKILHSVVARHSGR---FYTQGMHDVAAAL--ILLLV :  486
TgTBC13 : DKQAVQPLPFGV----PASH-MM--------YEYLRCVLEAYAVFRPDV---GYVQGMAYLAGAF--LLYM- :  621
TgTBC14 : YNAIEMDVHRTF----PSLP-FF----KEEGQTAMRHILQAYAVFDPEV---GYVQGMNFVVGTL--LHHSN :  181
TgTBC15 : -PEILRDVGRTF----PYRK-KFRE--AM--QQALERILRASANQLPKV---GYCQGMNFVAGVL--LEVLG :  147
TgTBC16 : ---------------------------DMTIGEGVRRLLQAYELYRPDI---GAVRGMDGLASVL--LCFM- :  512
TgTBC17 : FDDFNENSSRGYP---PSHE-FFRG--RCSIGAGIADVVECVVLHMPTV---GYVQGMAGIAGVL--LFFM- :  999
TgTBC18 : -DEIRRDVPRTF----PRHP-YFR---HSEGRAKLYAVLHAYACLCPST---GYCQGMNFLAGGL--LLFTG :  219
```

Supplementary Figure 1. Conservation of the TBC dual-finger active sites in TBC domain-containing candidates. The diagram presents the results of a multiple sequence alignment of TBC1-10, 12-18, showing only the relevant parts of the IxxDxxR and YxQ motifs. Residues highlighted in yellow indicate the dual-finger active sites. White residues on black background indicate 100% conservation, while white and black residues on grey background indicate 80% and 60% conservation, respectively.

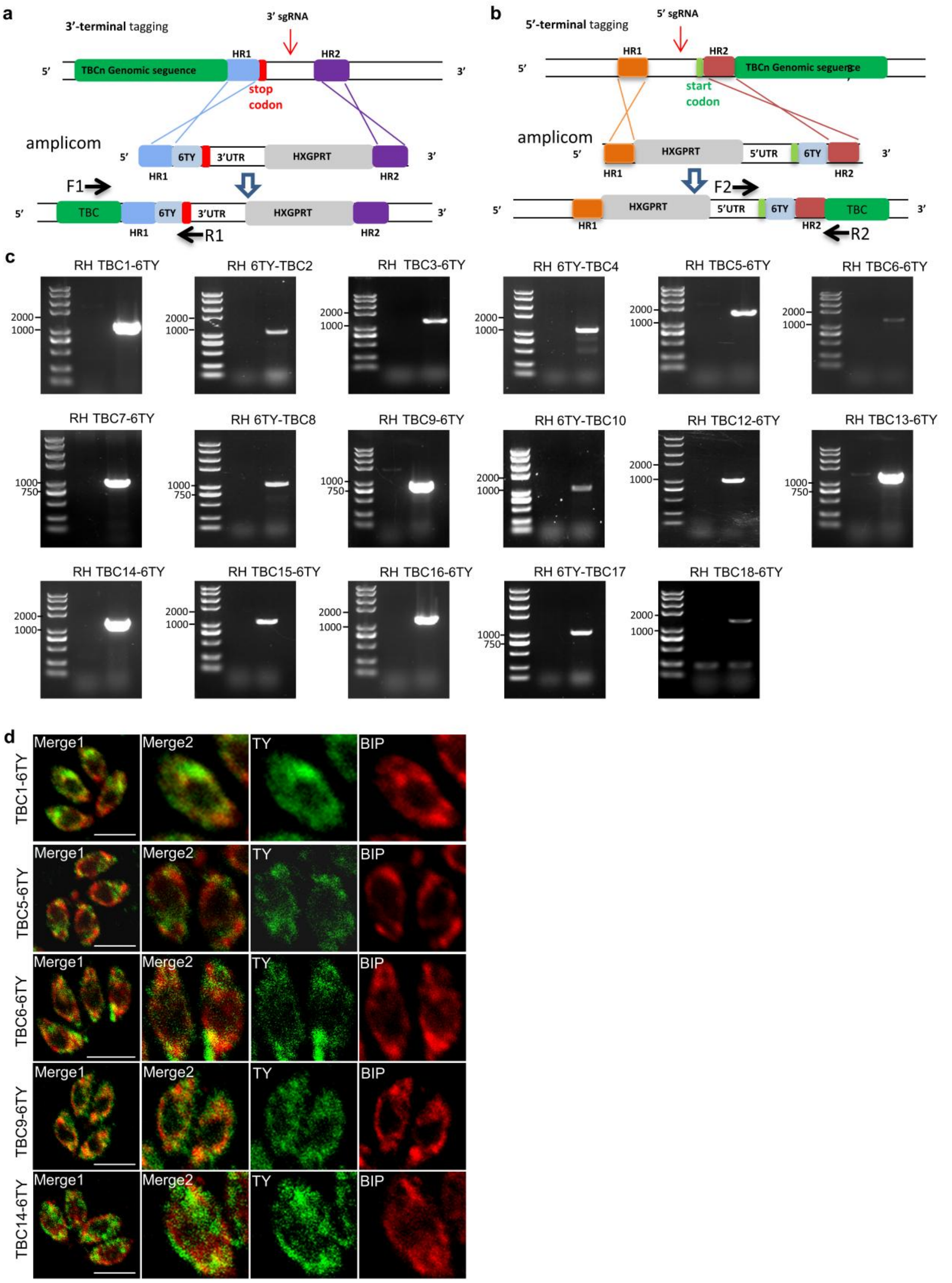

Supplementary Figure 2. Co-localization of some specific TBC proteins with the markers for the ER in *T. gondii*.

a Schematic of the strategy used to endogenously tag TBC1, 3, 5- 7, 9, 12-16, and 18 at the C-terminus in the parental line RHΔ*ku80*Δ*hxgprt* using a CRISPR/Cas9 approach.

b Schematic of the strategy used to endogenously tag TBC2, 4, 8, 10, and 17

at the N-terminus in RHΔ*ku80*Δ*hxgprt* using a CRISPR/Cas9 approach.

c Diagnostic PCR of the genetically modified lines. Primers (black arrows in **a** and **b**) were used to test genomic DNA from the parental (RH) and engineered lines. This PCR detected the gene integration of tagging amplicons in the engineered lines (750-2000bp).

d Confocal co-localization of the epitope Ty fused to TBC1, 5, 6, 9, and 14 (green) with the ER marker BIP (red), and zoom in on the ER region. Scale bar = 5 μm.

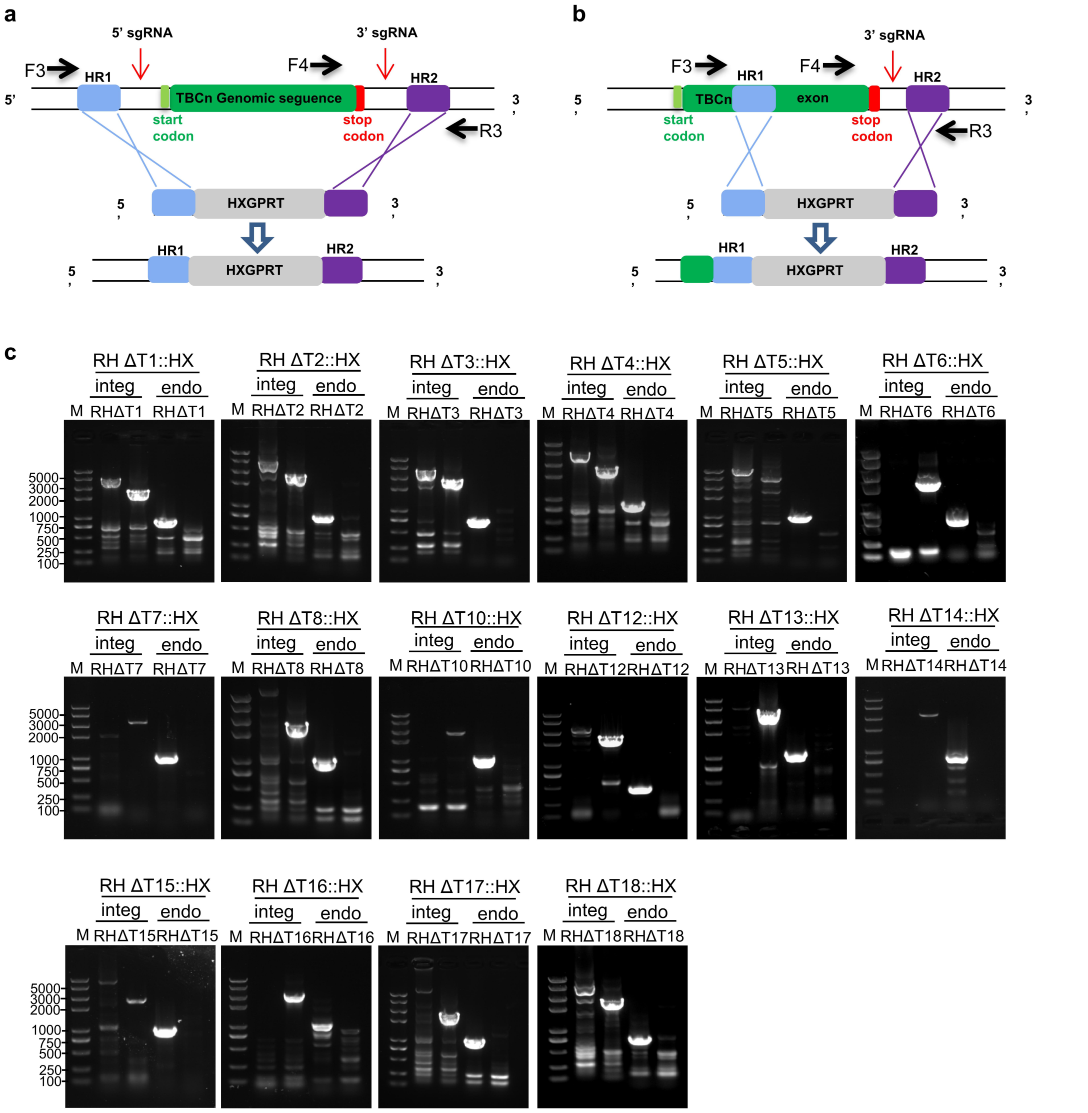

Supplementary Figure 3. Confirmation of knockout lines generated by CRISPR/Cas9.

a, **b** Schematics of the knockout generation and diagnostic PCR. TBC2, 4, 6-10, and 12-18 were deleted using a double sgRNA approach (**a**), while TBC1, 3 and 5 were deleted using a single sgRNA approach (**b**), as demonstrated in the diagrams.

c Diagnostic PCR of the knockout lines. The integration (integ) and endogenous (endo) PCRs were performed using primers illustrated in (**a**, **b**) (integ PCR primers: F3, R3; endo PCR primers: F4, R3). The integ PCR detected the gene integration in the targeted gene in the knockout lines (2-3kb), while the endo PCR detected the specific endogenous DNA in the RH line (400-1000bp).

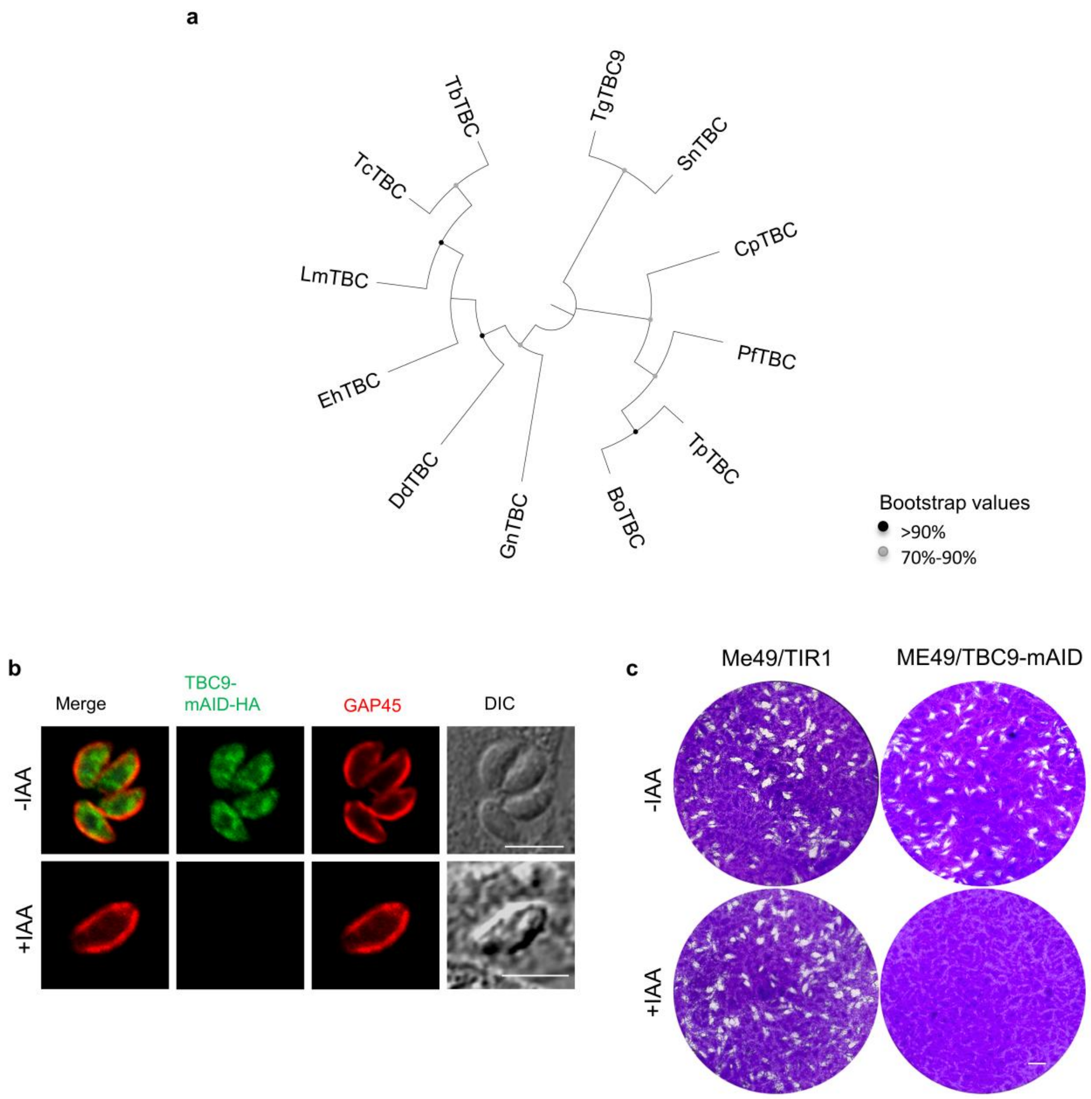

Supplementary Figure 4. TBC9 is ubiquitously present in parasites, and is essential in *T. gondii*.

a Evolutionary conservation of TBC9 in apicomplexan parasites and beyond. Phylogenetic tree based on the TBC domain of TBC9 and its orthologues from *Sarcocystis neurona* (*Sn*, SN3_01300335), *Cryptosporidium parvum* (*Cp*, cgd4_2130), *Plasmodium falciparum* (*Pf*, PF3D7_0904000), *Theileria parva* (*Tp*, TpMuguga_04g00105), *Babesia ovata* (*Bo*, BOVATA_008920), *Gregarina niphandrodes* (*Gn*, GNI_098330), *Dictyostelium discoideum* (*Dd*, Q54FT8), *Entamoeba histolytica* (*Eh*, EHI_009510), *Leishmania major* (*Lm*, LmjF.36.6370), *Trypanosoma cruzi* (*Tc*, C3747_216g36), and *Trypanosoma brucei* (*Tb*, Tb927.10.7680). Black spheres denote bootstrap values > 90%, and gray spheres denote bootstrap values between 70% and 90%.

b IFA of ME49/TBC9-mAID-3HA line in ± IAA treatment for 24 hours, using antibodies against GAP45 (red) and HA (green). Scale bar = 5 μm.

c Plaque formation of the ME49/TIR1 and ME49/TBC9-mAID lines. Parasites were grown on HFF monolayers in 6-well plates with ± IAA for 10 days, followed by fixation and staining with crystal violet. Scale bar = 2 mm.

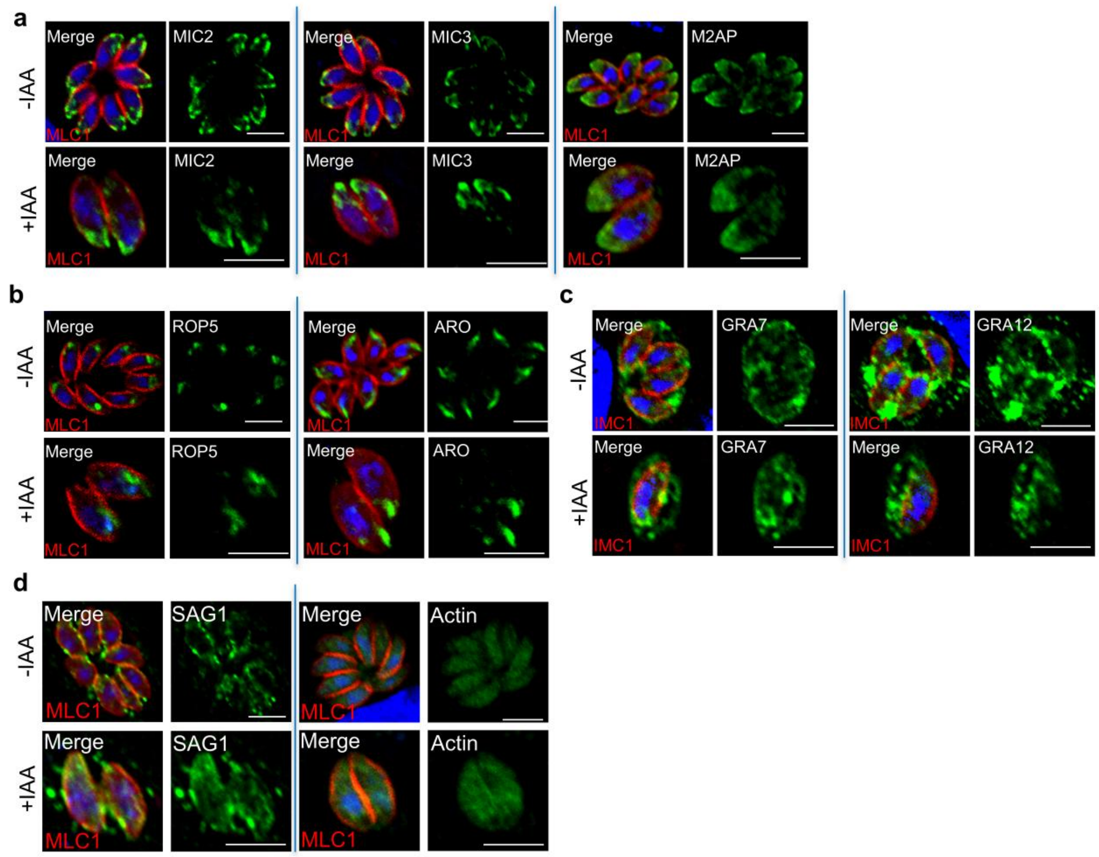

Supplementary Figure 5. The apical organelles and other structures appeared to be weakly affected upon depletion of TBC9.

a, **b** TBC9-mAID parasites were grown on HFF monolayers for 16 hours, followed by ± IAA treatment for 13 hours. IFA was performed using antibodies against the microneme proteins MIC2, MIC3, and MIAP (green) (**a**), and rhoptry proteins ROP5 and ARO (green) (**b**).

c TBC9-mAID parasites were grown on HFF monolayers for 6 hours, followed by ± IAA treatment for 16 hours before processing for IFA analyses using the anti-GRA7 and anti-GRA12 antibodies (green).

d TBC9-mAID parasites were grown on HFF monolayers for 16 hours, followed by ± IAA treatment for 13 hours. IFA was performed using antibodies against the surface antigen 1 (SAG1, green), actin (Actin, green), and MLC1 (red). Hoechst (blue) stained nuclei. Scale bar = 5 μm.

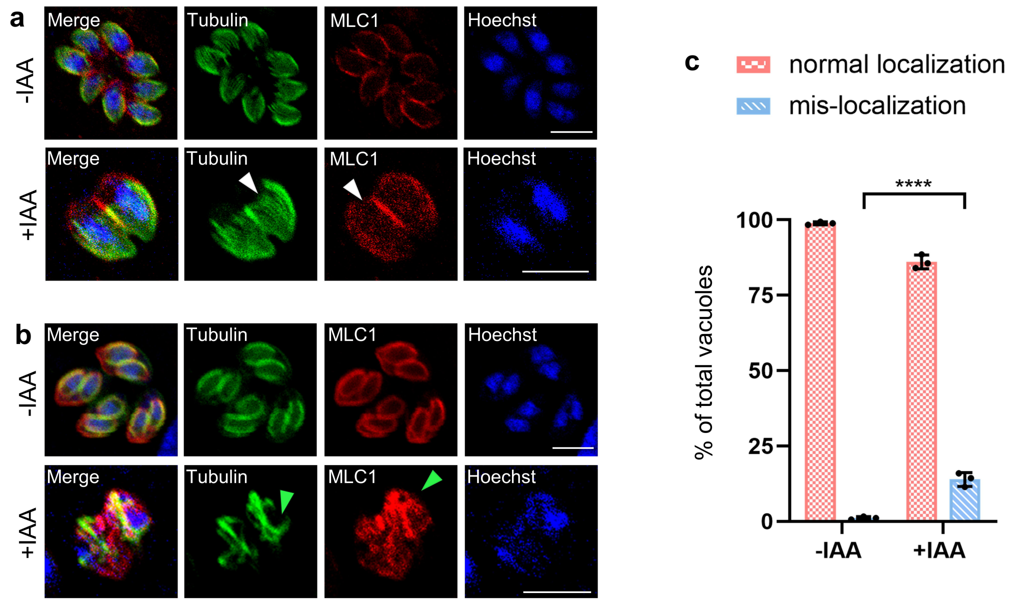

Supplementary Figure 6. Depletion of TBC9 affects the cytoskeleton of *T. gondii* during daughter IMC formation.

a, **b** TBC9-mAID parasites were grown on HFF monolayers for 16 hours, followed by ± IAA treatment for 13 hours before processing for IFA analyses. Hoechst (blue) dye was used to stain the nucleus. Using antibody combinations against MLC1 (red) and Tubulin (green), IFA results were obtained for morphologically normal parasites without daughter bud formation (**a**, white arrowheads) and morphologically fragmented parasites with daughter bud formation (**b**, green arrowheads). Scale bar = 5 μm.

c Vacuoles with normal localization or mis-localization of Tubulin were counted (≥200 vacuoles for each replicate). Data (N=3 independent experiments; n=3 replicates) are presented as the means ± SD, and two-way ANOVA with Sidak's multiple comparison test was performed. (****, $p < 0.0001$).

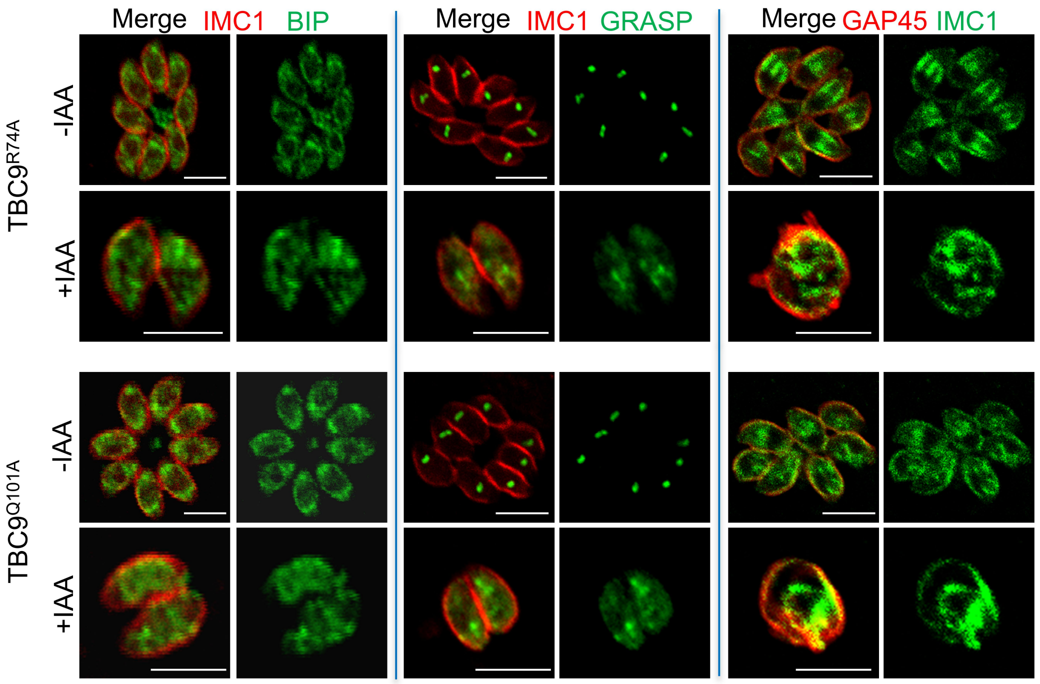

Supplementary Figure 7. Mislocalization of ER, Golgi, and IMC1 in TBC9-mAID-3xHA line complemented with TBC9 mutants upon TBC9 depletion. IFA detection of the TBC9-mAID-3xHA line complemented with TBC9^{R74A}-2TY or TBC9^{Q101A}-2TY. Parasites were grown on HFF monolayers for 16 hours, followed by ± IAA treatment for 13 hours before processing for IFA analyses using antibodies against the proteins indicated in the images: BIP for the ER; GRASP for the Golgi; GAP45 and IMC1 for the inner membrane complex. Scale bar = 5 μm.

Supplementary Table 1. Information summary of 17 TBC-domain containing proteins in *T. gondii*. The candidates were identified by the InterPro ID IPR035969 (Rab-GAP-TBC domain superfamily) as query in the ToxoDB. The phenotype scores were retrieved from a CRISPR-library screening[1], while the dual finger sites in the TBC domain were predicted by the multiple sequence alignment. The protein localization was derived from the tagging and IFA analyses, as described in this study.

TBC protein	ToxoDB-ID	Phenotype Score	Protein Size (kDa)	Localization (Tachyzoite)	IxxDxxR or YxQ
TBC1	TGGT1_275350	-0.58	102	ER	R&Q
TBC2	TGGT1_274130	-1.84	227	TGN	Q
TBC3	TGGT1_218870	-0.74	53	TGN; Cytoplasmic Vesicles	R&Q
TBC4	TGGT1_285730	-0.36	49	Cytoplasmic Vesicles	R&Q
TBC5	TGGT1_250680	-0.06	207	ER	×
TBC6	TGGT1_237280	-0.16	233	ER	R
TBC7	TGGT1_216430	0.09	358	Not expressed	R&Q
TBC8	TGGT1_261200	-1	100	Cytoplasmic Vesicles	R&Q
TBC9	TGGT1_226550	-4.82	36	ER	R&Q
TBC10	TGGT1_203910	0.53	229	Cytoplasmic Vesicles ; residual body	R&Q
TBC12	TGGT1_223640	-0.6	178	Cytoplasmic Vesicles	R&Q
TBC13	TGGT1_221710	-2.04	182	Cytoplasmic Vesicles	Q
TBC14	TGGT1_289820	-1.14	114	ER	R&Q
TBC15	TGGT1_226850	0.54	118	Daughter IMC	R&Q
TBC16	TGGT1_312300	-1.19	324	Cytoplasmic Vesicles	×
TBC17	TGGT1_266830	0.3	426	Cytoplasmic Vesicles	R&Q
TBC18	TGGT1_213325	-2.61	293	TGN	R&Q

Supplementary Reference

1 Sidik, S. M. *et al.* A Genome-wide CRISPR Screen in *Toxoplasma* Identifies Essential Apicomplexan Genes. *Cell* **166**, 1423-1435.e1412 (2016).
