# Supplementary material for: TBC9, an essential TBC-domain protein, regulates early vesicular transport and IMC formation in Toxoplasma gondii
Source: Commun Biol. 2024 May 18;7:596. doi: 10.1038/s42003-024-06310-6 (PMC11102469; doi:10.1038/s42003-024-06310-6)
Supplement: Supplementary file 3 — Description of Additional Supplementary Files [file 42003_2024_6310_MOESM3_ESM.pdf]

Description of Additional Supplementary Files

File name: Supplementary Data 1

Description: Lines, plasmids and primers used in this study.

File name: Supplementary Data 2

Description: Mass-spectrometry datasets generated for the immuno-precipitated samples with the TBC9-6HA and parental lines of T. gondii.

File name: Supplementary Data 3

Description: The source data behind the graphs in the paper
