# Supplementary material for: TBC9, an essential TBC-domain protein, regulates early vesicular transport and IMC formation in Toxoplasma gondii
Source: Commun Biol. 2024 May 18;7:596. doi: 10.1038/s42003-024-06310-6 (PMC11102469; doi:10.1038/s42003-024-06310-6)
Supplement: Supplementary file 7 — Reporting Summary [file 42003_2024_6310_MOESM7_ESM.pdf]

nature portfolio

Corresponding author(s): Shaojun Long

Last updated by author(s): Apr 26, 2024

# Reporting Summary

Nature Portfolio wishes to improve the reproducibility of the work that we publish. This form provides structure for consistency and transparency in reporting. For further information on Nature Portfolio policies, see our Editorial Policies and the Editorial Policy Checklist.

## Statistics

For all statistical analyses, confirm that the following items are present in the figure legend, table legend, main text, or Methods section.

| n/a | Confirmed | |
|---|---|---|
| ☐ | ☒ | The exact sample size ($n$) for each experimental group/condition, given as a discrete number and unit of measurement |
| ☐ | ☒ | A statement on whether measurements were taken from distinct samples or whether the same sample was measured repeatedly |
| ☐ | ☒ | The statistical test(s) used AND whether they are one- or two-sided<br>*Only common tests should be described solely by name; describe more complex techniques in the Methods section.* |
| ☒ | ☐ | A description of all covariates tested |
| ☐ | ☒ | A description of any assumptions or corrections, such as tests of normality and adjustment for multiple comparisons |
| ☒ | ☐ | A full description of the statistical parameters including central tendency (e.g. means) or other basic estimates (e.g. regression coefficient) AND variation (e.g. standard deviation) or associated estimates of uncertainty (e.g. confidence intervals) |
| ☒ | ☐ | For null hypothesis testing, the test statistic (e.g. $F$, $t$, $r$) with confidence intervals, effect sizes, degrees of freedom and $P$ value noted<br>*Give P values as exact values whenever suitable.* |
| ☒ | ☐ | For Bayesian analysis, information on the choice of priors and Markov chain Monte Carlo settings |
| ☒ | ☐ | For hierarchical and complex designs, identification of the appropriate level for tests and full reporting of outcomes |
| ☒ | ☐ | Estimates of effect sizes (e.g. Cohen's $d$, Pearson's $r$), indicating how they were calculated |

*Our web collection on statistics for biologists contains articles on many of the points above.*

## Software and code

Policy information about availability of computer code

| | |
|---|---|
| Data collection | Proteomics data: Raw data from the mass spectrometer were preprocessed by PeaksOnline engine for peak picking<br>Microscopy: Nikon Confocal C2+ and NIS Elements AR (NIKON)<br>DNA and protein gels: BIO-RAD ChemiDoc MP Imaging System |
| Data analysis | Image J version 1.52p, Graphpad Prism software (version 8.4.0), Nikon Confocal C2+ and NIS Elements AR 5.11.01 (NIKON). The L-INS-I algorithm in Mafft software (v7.490, Kyoto Japan) for sequence alignment; spurious sequences or poorly arranged regions were automatically removed using trimAL software (https://vicfero.github.io/trimal/); the Le-Gascuel model was used to construct the phylogenetic tree through FastTree (v2.1, Berkeley, CA, USA). |

For manuscripts utilizing custom algorithms or software that are central to the research but not yet described in published literature, software must be made available to editors and reviewers. We strongly encourage code deposition in a community repository (e.g. GitHub). See the Nature Portfolio guidelines for submitting code & software for further information.

# Data

Policy information about availability of data

All manuscripts must include a data availability statement. This statement should provide the following information, where applicable:

- Accession codes, unique identifiers, or web links for publicly available datasets
- A description of any restrictions on data availability
- For clinical datasets or third party data, please ensure that the statement adheres to our policy

The proteomic data reported in this paper have been deposited in the OMIX, China National Center for Bioinformation/Beijing Institute of Genomics, Chinese Academy of Sciences (https://ngdc.cncb.ac.cn/omix: accession no. OMIX005138). Minimally processed data of the proteomics are available in Supplementary Data 2. The source data behind the graphs in the paper is available in Supplementary Data 3.

# Research involving human participants, their data, or biological material

Policy information about studies with human participants or human data. See also policy information about sex, gender (identity/presentation), and sexual orientation and race, ethnicity and racism.

| | |
|---|---|
| Reporting on sex and gender | This study did not involve human participants. |
| Reporting on race, ethnicity, or other socially relevant groupings | This study did not involve human participants. |
| Population characteristics | This study did not involve human participants. |
| Recruitment | This study did not involve human participants. |
| Ethics oversight | This study did not involve human participants. |

Note that full information on the approval of the study protocol must also be provided in the manuscript.

# Field-specific reporting

Please select the one below that is the best fit for your research. If you are not sure, read the appropriate sections before making your selection.

☒ Life sciences ☐ Behavioural & social sciences ☐ Ecological, evolutionary & environmental sciences

For a reference copy of the document with all sections, see nature.com/documents/nr-reporting-summary-flat.pdf

# Life sciences study design

All studies must disclose on these points even when the disclosure is negative.

| | |
|---|---|
| Sample size | Three independent experiments were performed with triplicates for each, which add up the sample sizes to 9 in the study. |
| Data exclusions | No data were excluded from the analyses. |
| Replication | Three replicates were performed in each independent biological experiment, and three independent experiments were performed. All attempts at replication were successful. |
| Randomization | The quantificaiton under microscope was performed for randomly selected fields, and over 150 vacuoles or parasites were scored. |
| Blinding | The investigators were blinded to group allocation during data collection and/or analysis. |

# Reporting for specific materials, systems and methods

We require information from authors about some types of materials, experimental systems and methods used in many studies. Here, indicate whether each material, system or method listed is relevant to your study. If you are not sure if a list item applies to your research, read the appropriate section before selecting a response.

## Materials & experimental systems

| n/a | Involved in the study |
| --- | --- |
| ☐ | ☒ Antibodies |
| ☐ | ☒ Eukaryotic cell lines |
| ☒ | ☐ Palaeontology and archaeology |
| ☐ | ☒ Animals and other organisms |
| ☒ | ☐ Clinical data |
| ☒ | ☐ Dual use research of concern |
| ☒ | ☐ Plants |

## Methods

| n/a | Involved in the study |
| --- | --- |
| ☒ | ☐ ChIP-seq |
| ☒ | ☐ Flow cytometry |
| ☒ | ☐ MRI-based neuroimaging |

# Antibodies

| | |
| --- | --- |
| Antibodies used | Primary antibodies, including mouse anti-HA (BioLegend, #901501), rabbit anti-HA (Thermo Fisher Scientific, #71-5500), mouse anti-his (Abmart, M30111F), and rabbit anti-MBP (Yesen, 31201ES20) were commercially purchased. Rabbit anti-GRA12, rabbit anti-ARO, rabbit anti-MIC3 and rabbit anti-MIAP were obtained from other research laboratories. Other primary antibodies including mouse anti-Ty (BB2), rabbit anti-ROP5 (51), mouse mAb 6D10 anti-MIC2 (53), mouse mAb DG52 anti-SAG1, rabbit anti-GRA7 (56) were generous gifts. Primary antibodies made in this laboratory include mouse anti-IMC1, rabbit anti-GAP45, rabbit anti-HSP60, rabbit anti-ACP, rabbit anti-Actin, mouse anti-MLC1, rabbit anti-STX6, rabbit anti-IPPS, Rabbit anti-BIP, rabbit anti-Tubulin, rabbit anti-Centrin1, and mouse anti-GRASP. The secondary antibodies included anti-mouse antibodies conjugated with Alexa Fluors 488/568 (Thermo Fisher Scientific, A-11001 and A-11004), anti-rabbit antibodies conjugated with Alexa Fluors 488/568 (Thermo Fisher Scientific, A-11008 and A-11011), Alexa Fluor 488-Streptavidin (Thermo Fisher Scientific, S11223), and fluorescent reagents conjugated with LI-COR 680 CW and 800 CW (LICOR, 926-32210, 926-32211, 926-68071 and 926-68070). |
| Validation | All commercially purchased primary antibodies have validation statements on the manufacturer's website. Mouse anti-IMC1, rabbit anti-GAP45, rabbit anti-HSP60, rabbit anti-ACP, rabbit anti-Actin, mouse anti-MLC1, rabbit anti-STX6 and rabbit anti-IPPS antibodies were generated in our recent studies (DOI: 10.1038/s41467-023-36571-4; DOI: 10.1128/mbio). Rabbit anti-BIP, rabbit anti-Tubulin, rabbit anti- Centrin1, and mouse anti- GRASP were generated in this study. Primary antibodies from other laboratories have been used in previous studies and are cited in our manuscript in relevant part of the material and methods. |

# Eukaryotic cell lines

Policy information about cell lines and Sex and Gender in Research

| | |
| --- | --- |
| Cell line source(s) | Human foreskin fibroblast HFF-1 cells (ATCC, SCRC-1041) |
| Authentication | The cell line was purchased and permitted to used in the lab for supporting the parasite cultivation. |
| Mycoplasma contamination | All cell lines tested negative for mycoplasma contamination. |
| Commonly misidentified lines (See ICLAC register) | No commonly misidentified lines were used in this study. |

# Animals and other research organisms

Policy information about studies involving animals; ARRIVE guidelines recommended for reporting animal research, and Sex and Gender in Research

| | |
| --- | --- |
| Laboratory animals | BALB/C mice; New Zealand white Rabbit |
| Wild animals | This study did not involve wild animals. |
| Reporting on sex | The animals' sex was not limited. |
| Field-collected samples | This study did not involve samples collected from the field. |
| Ethics oversight | The mouse and rabbit experiments were conducted according to the guidelines and regulations issued by the Veterinary Office of Chain Agricultural University ( Issue No. AW11402202-2-1). |

Note that full information on the approval of the study protocol must also be provided in the manuscript.

## Plants

| | |
|---|---|
| Seed stocks | This study did not involve seed. |
| Novel plant genotypes | This study did not involve plants. |
| Authentication | This study did not involve plants. |
